# Effect of Discharge Gas Composition on SiC Etching in an HFE-347mmy/O_2_/Ar Plasma

**DOI:** 10.3390/ma17163917

**Published:** 2024-08-07

**Authors:** Sanghyun You, Eunjae Sun, Heeyeop Chae, Chang-Koo Kim

**Affiliations:** 1 Department of Chemical Engineering and Department of Energy Systems Research, Ajou University, Worldcup-ro 206, Yeongtong-gu, Suwon 16499, Republic of Korea; you15717@ajou.ac.kr (S.Y.); ejsun2@ajou.ac.kr (E.S.); 2 School of Chemical Engineering, Sungkyunkwan University (SKKU), Seobu-ro 2066, Jangan-gu, Suwon 16419, Republic of Korea; hchae@skku.edu

**Keywords:** plasma etching, SiC, global warming potential, HFE-347mmy, etch rate

## Abstract

This study explores the impact of varying discharge gas compositions on the etching performance of silicon carbide (SiC) in a heptafluoroisopropyl methyl ether (HFE-347mmy)/O_2_/Ar plasma. SiC is increasingly favored for high-temperature and high-power applications due to its wide bandgap and high dielectric strength, but its chemical stability makes it challenging to etch. This research explores the use of HFE-347mmy as a low-global-warming-potential (GWP) alternative to the conventional high-GWP fluorinated gasses that are typically used in plasma etching. By examining the behavior of SiC etch rates and analyzing the formation of fluorocarbon films and Si-O bonds, this study provides insights into optimizing plasma conditions for effective SiC etching, while addressing environmental concerns associated with high-GWP gasses.

## 1. Introduction

Silicon (Si) semiconductor devices are used in various electronic devices. However, they face problems operating at high temperatures and high voltages, owing to their narrow bandgap (1.1 eV) and low dielectric strength (0.3 MV/cm), resulting in a high power loss and low switching frequencies [1]. Accordingly, several materials are being investigated as an alternative to Si. Among them, silicon carbide (SiC) possesses advantageous physical properties such as a wide bandgap (3.2 eV) and a high dielectric strength (3 MV/cm), rendering it suitable for high-temperature, high-power, and high-frequency applications [2,3,4].

The etching of SiC using wet chemical etching is constrained by its outstanding chemical stability. Typically, the wet etching of SiC is achieved either in KOH solutions at elevated temperatures (>600 °C) [1] or with photoelectrochemical etching [5,6], which complicates feature-size control. Consequently, dry etching in a gaseous plasma is commonly employed for SiC etching.

During plasma etching, a substrate is etched using a reaction with reactive radicals generated from a plasma, forming volatile products. Therefore, plasmas containing fluorinated gasses (such as CHF_3_, CF_4_, and SF_6_) mixed with O_2_ are typically used for SiC etching because F and O radicals react with Si or C to produce volatile SiF_4_, CF_4_, CO, and CO_2_ [7,8,9,10,11,12,13]. However, these fluorinated gasses are problematic from an environmental viewpoint because of their high global warming potentials (GWPs) (CHF_3_: 14,600, CF_4_: 7300, and SF_6_: 25,200). They have been categorized as greenhouse gasses under the Kyoto Protocol, and reduction targets for greenhouse gas emissions have been set for each country in the Paris Agreement. To address this issue, efforts have been made to reduce emissions of high-GWP gasses by process optimization, abatement, and recovery/capture methods. These methods have the advantage of reducing high-GWP gas emissions under the use of existing processes. However, the European Union is currently seeking to completely restrict the use of per- and polyfluoroalkyl substances (PFASs), and the United States has also announced the strengthening of individual regulations on PFASs [14,15]. Since high-GWP fluorinated gasses used in SiC etching are not free from PFAS regulations, it is effective to replace them with low-GWP alternatives rather than reducing high-GWP gas emissions by process optimization, abatement, and recovery/capture.

The development of alternative chemistries as substitutes for high-GWP fluorinated gasses has been challenged for the plasma etching of dielectrics such as SiO_2_ and Si_3_N_4_ [16,17,18,19]. Several types of alternative chemistries to high-GWP fluorinated gasses have been examined for dielectric etching, including unsaturated fluorocarbons [20,21], iodofluorocarbons [22,23], hydrofluoroethers [24,25,26], and hydrofluoroalcohols [27]. However, the evaluation of low-GWP materials for SiC etching has rarely been reported.

Recently, we reported the use of heptafluoroisopropyl methyl ether (HFE-347mmy, CF_3_CFOCH_3_CF_3_) as a low-GWP alternative for SiC etching [28], demonstrating higher etch rates and smoother etched surfaces compared to traditional gasses such as SF_6_. HFE-347mmy belongs to a group of fluorinated ethers and contains an oxygen atom. It has been used for the plasma etching of SiO_2_ [29]. The oxygen atoms in fluorinated ethers may generate oxygen radicals and ions in the plasma, aiding in SiO_2_ etching.

As an extension of the previous study, the effect of plasma chemistry on SiC etching in an HFE-347mmy/O_2_/Ar plasma was investigated in the present article. The behavior of the SiC etch rates with respect to the composition of discharge gasses in the HFE-347mmy/O_2_/Ar plasma was studied in terms of the relative amounts of radicals generated from the plasma, the thickness of the steady-state fluorocarbon films formed on the SiC surface, and the fraction of Si-O bonds occupying the SiC surface.

## 2. Materials and Methods

**Figure 1:**
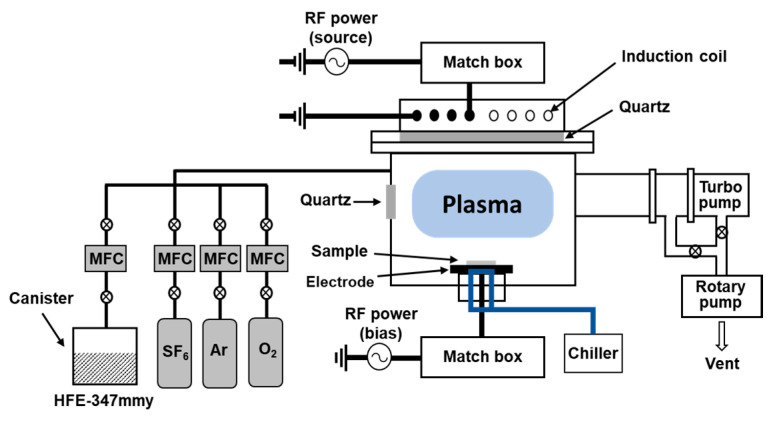
Schematic diagram of an inductively coupled plasma system for SiC etching.

SiC etching was performed in an inductively coupled plasma (ICP) system, as shown in Figure 1. Two 13.56 MHz radio-frequency power generators were used separately to supply source and bias powers. The source power was applied through a five-turn induction coil to ignite a plasma. The induction coil had a planar shape and was placed on top of a quartz window. The bias power was applied with a stainless-steel electrode to bias a specimen on the electrode. The electrode was kept at 15 °C using a chiller. The quartz window on the sidewall of the ICP chamber allowed us to check the optical properties of the plasma.

The discharge gas was a mixture of HFE-347mmy/O_2_/Ar. HFE-347mmy was in a liquid phase at room temperature, owing to its boiling point of 29 °C. It was vaporized in a canister, which was heated to 75 °C. The vaporized HFE-347mmy was mixed with O_2_ and Ar prior to its entry into the ICP chamber, and the resultant gas mixture was then introduced into the chamber. Table 1 shows the typical properties of HFE-347mmy.

HFE-347mmy/O_2_/Ar was used for SiC etching. The flow rate of Ar was fixed at 5 sccm. The flow rates of HFE-347mmy and O_2_ were varied from 1 to 9 sccm to maintain a total flow rate of 15 sccm. For example, when the flow rate of HFE-347mmy was 1 sccm, the flow rate of O_2_ was 9 sccm, and vice versa. Other process conditions were fixed as follows: chamber pressure = 4.0 Pa, source power = 500 W, bias voltage = −500 V, and electrode temperature = 15 °C. The specimen was an n-type 4H-SiC substrate. Each specimen was in a rectangular shape (10 × 5 mm^2^).

The etch rates of SiC were determined by measuring the changes in the thickness of the SiC substrate with a surface profiler (Ambios Technology, XP-1, Santa Cruz, CA, USA) after the etching process. The relative amounts of radicals generated in the plasma were obtained using optical emission spectroscopy (OES, Avantes, AvaSpec-ULS2048-USB2-RM, Apeldoorn, The Netherlands). An analysis of the thickness of a steady-state fluorocarbon film formed on the SiC surface was performed using X-ray photoelectron spectroscopy (XPS, Thermo Electron, K-Alpha, Waltham, MA, USA), which had an X-ray source of 1486.6 eV generated from a movable Al anode at 15 kV.

## 3. Surface Reaction Mechanisms in Plasma Etching of SiC

**Figure 2:**
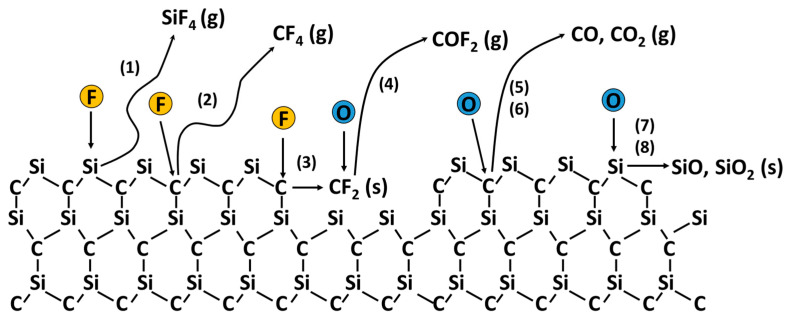
Schematic of the surface reaction mechanism for SiC etching in a plasma containing fluorinated gasses and oxygen. The numbers in parentheses represent the equation numbers given in the text.

Figure 2 illustrates the surface reaction mechanism involved in SiC etching. Typically, SiC is etched in plasmas containing fluorinated gasses such as SF_6_, CF_4_, and CHF_3_ mixed with O_2_. The major reactions during SiC etching are as follows [30]:

Si + 4F → SiF_4_ (g) [removal of F], (1)

C + 4F → CF_4_ (g)   [removal of C], (2)

C + 2F → CF_2_ (s)   [formation of CF_2_], (3)

O + CF_2_ → COF_2_ (g)   [removal of C], (4)

C + O → CO (g)   [removal of C],(5)

C + 2O → CO_2_ (g)   [removal of C], (6)

Si + O → SiO (s)   [formation of SiO],(7)

Si + 2O → SiO_2_ (s)   [formation of SiO_2_], (8)

F radicals generated from fluorinated gasses react with Si and C on SiC surfaces, forming volatile products SiF_4_ and CF_4_ (Equations (1) and (2)). Additionally, the reaction of F radicals with C produces CF_2_ (Equation (3)), serving as a precursor for the formation of fluorocarbon films on SiC surfaces. The fluorocarbon films act as an etch barrier against radicals and ions. Simultaneously, the fluorocarbon films are consumed by the reaction with O radicals generated from O_2_, forming volatile products of COF_2_ (Equation (4)).

O radicals can either facilitate or hinder SiC etching, depending on their reaction with Si or C. The reaction of O radicals with C on the SiC surface results in volatile products such as CO and CO_2_ (Equations (5) and (6)), leading to SiC etching. However, O radicals reacting with Si produce SiO and SiO_2_ on the SiC surface (Equations (7) and (8)). The formation of SiO and SiO_2_ reduces the active Si sites for the reaction with F, resulting in the suppression of SiC etching.

## 4. Results and Discussion

**Figure 3:**
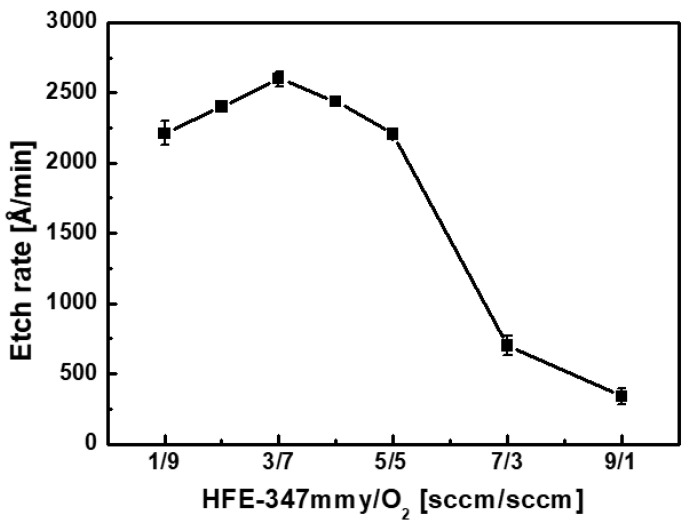
Etch rate of SiC in the HFE-347mmy/O_2_/Ar plasma at various flow rates of HFE-347mmy/O_2_.

Figure 3 shows the etch rate of SiC in the HFE-347mmy/O_2_/Ar plasma at various flow rates of HFE-347mmy/O_2_. The etch rate increased from 2210 to 2600 Å/min with an increase in the HFE-347mmy/O_2_ ratio from 0.11 (i.e., HFE-347mmy/O_2_ = 1/9 sccm) to 0.43 (HFE-347mmy/O_2_ = 3/7 sccm). The etch rate of SiC reached the maximum at an HFE-347mmy/O_2_ ratio of 0.43 and then decreased with the further increase in the HFE-347mmy/O_2_ ratio. The etch rate was as low as 340 Å/min when the HFE-347mmy/O_2_ ratio was 9.0 (HFE-347mmy/O_2_ = 9/1 sccm).

**Figure 4:**
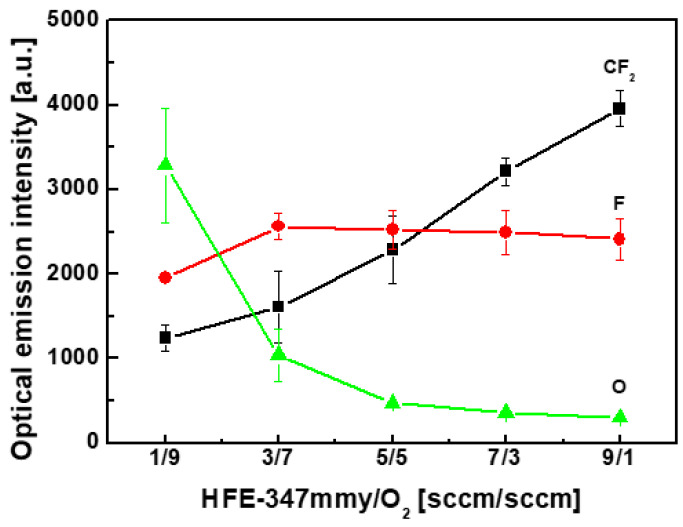
Optical emission intensity of F, CF_2_, and O peaks in the HFE-347mmy/O_2_/Ar plasma at various flow rates of HFE-347mmy/O_2_.

To investigate the behavior of the SiC etch rate with respect to the HFE-347mmy/O_2_ ratio, the relative amounts of radicals produced in the HFE-347mmy/O_2_/Ar plasma were obtained using OES. Figure 4 shows the optical emission intensity of the F, CF_2_, and O peaks in the HFE-347mmy/O_2_/Ar plasma at various flow rates of HFE-347mmy/O_2_. The discharge condition for the OES measurements was the same as that for SiC etching. As the HFE-347mmy/O_2_ ratio increased, the CF_2_ peak intensity increased, while the O-peak intensity decreased. This corresponded to the changes in the flow rates of HFE-347mmy and O_2_, which were the parent materials for CF_2_ and O radicals, respectively. In particular, the CF_2_ peak intensity increased with the increasing flow rate of HFE-347mmy, and the O-peak intensity increased with increasing O_2_ flow rate. The change in the F-peak intensity with the flow rates of HFE-347mmy/O_2_ exhibited the same behavior as that in the etch rate of SiC. The F peak intensity increased with the initial increase in the HFE-347mmy/O_2_ ratio, reaching a maximum at an HFE-347mmy/O_2_ ratio of 0.43 (HFE-347mmy/O_2_ = 3/7 sccm), and decreased with the further increase in the HFE-347mmy/O_2_ ratio.

Although the intensities of the F and O peaks (correspondingly, the relative amounts of F and O radicals, which are the main etchants for SiC etching) decreased for HFE-347mmy/O_2_ ratios higher than 0.43, such a variation cannot solely explain the large decrease in the SiC etch rate in this HFE-347mmy/O_2_ ratio regime. As mentioned earlier, the fluorocarbon films are formed on the SiC surfaces during the plasma etching of SiC in fluorinated gasses. These fluorocarbon films prevent radicals or ions from arriving at the underlying substrate and suppress the etching of SiC. Therefore, the etch rate of SiC is affected by the thickness of the steady-state fluorocarbon film.

**Figure 5:**
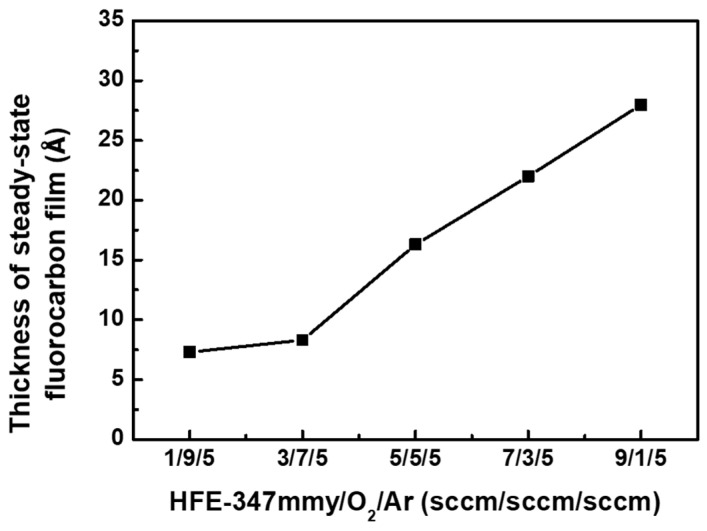
Thickness of the steady-state fluorocarbon films formed on the SiC surfaces in the HFE-347mmy/O_2_/Ar plasma at various flow rates of HFE-347mmy/O_2_.

The thickness of the steady-state fluorocarbon films was obtained by comparing the XPS intensities of Si 2p spectra before and after the etching process [31]. XPS measurements were conducted on the SiC surfaces that had been etched for 1 min. Figure 5 shows the thickness of the steady-state fluorocarbon films formed on the SiC surfaces in the HFE-347mmy/O_2_/Ar plasma at various flow rates of HFE-347mmy/O_2_. The film thickness slightly increased with the initial increase in the HFE-347mmy/O_2_ ratio and then rapidly increased at ratios higher than 0.43 (HFE-347mmy/O_2_ = 3/7 sccm). This agreed with the change in the optical emission intensity of the CF_2_ peak with the HFE-347mmy/O_2_ ratio (see Figure 4) because CF_2_ radicals are the main precursor for the deposition of the fluorocarbon film. Since a thicker fluorocarbon film leads to a slower etch rate, a large decrease in the SiC etch rate at HFE-347mmy/O_2_ ratios higher than 0.43 is attributed to both a decrease in the amounts of F and O radicals and an increase in the thickness of the steady-state fluorocarbon film.

As shown in Figure 2, SiC is etched by the reactions of not only F, but also O radicals with the substrate. Specifically, the higher the amount of O radicals present, the more SiC is etched. However, when the O_2_ flow rate increased from 7 to 9 sccm, the SiC etch rate decreased, even if the amount of O radicals increased (see Figure 3 and Figure 4). Obviously, it can be argued that the etch rate may decrease because of a decrease in the amount of F radicals in this flow rate regime. However, as the increase in the amount of O radicals is much greater than the decrease in that of F radicals, the SiC etch rate is expected to increase.

The decrease in the SiC etch rate with the O_2_ flow rate increasing from 7 to 9 sccm can be explained by the formation of SiO and SiO_2_ with an excess amount of O radicals. When there are sufficient O radicals in the plasma at high flow rates of O_2_, the reactions of O with Si rather than C play a major role, forming SiO and SiO_2_ on the SiC surfaces. The formation of SiO and SiO_2_ reduces the reaction probability of Si with F, causing the suppression of SiC etching. Under the process conditions used in this study, the etch rate of SiC decreased at O_2_ flow rates higher than 7 sccm, possibly because the formation of SiO and SiO_2_ was dominant over SiC etching. This explanation is plausible considering the reaction enthalpies for the formation of SiO_2_ and the removal of C (or SiC etching). The standard enthalpy for the formation of SiO_2_ (Equation (8)) is −1404 kJ·mol^−1^. In contrast, the standard enthalpies for the removal of C (Equations (4)–(6)) are −746 kJ·mol^−1^, −361 kJ·mol^−1^, and −888 kJ·mol^−1^, respectively. Thus, it can be said that the formation of SiO_2_ is more favorable than SiC etching when there are sufficient O radicals. To support the effect of the formation of the Si-O bond on the SiC etch rate, the Si-O occupancy is defined as the fraction of Si-O bonds occupying the SiC surface and is calculated from the XPS measurements using Equation (9):

(9) $$\text{Si-O}\;\mathrm{occupancy}=\frac{\mathrm{XPS}\;\mathrm{peak}\;\mathrm{area}\;\mathrm{of}\;\text{Si-O}}{\mathrm{XPS}\;\mathrm{peak}\;\mathrm{area}\;\mathrm{of}\;\text{Si-O}\;\mathrm{and}\;\text{Si-C}}.$$

**Figure 6:**
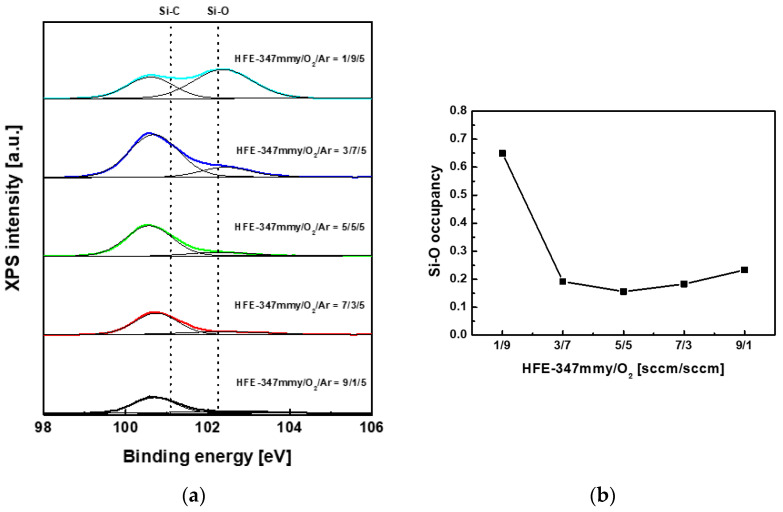
(**a**) Si 2p XPS spectra of SiC etched in the HFE-347mmy/O_2_/Ar plasma and (**b**) Si-O occupancy at various flow rates of HFE-347mmy/O_2_. The thin lines in (**a**) denote the deconvolution of the spectra.

Figure 6 presents the Si 2p XPS spectra of SiC etched in the HFE-347mmy/O_2_/Ar plasma, as well as the Si-O occupancy determined from the spectra at various flow rates of HFE-347mmy/O_2_. As the O_2_ flow rate increased from 1 to 7 sccm (from right to left in Figure 6b), the variation in the Si-O occupancy was negligible. However, the Si-O occupancy greatly increased with an increase in the O_2_ flow rate from 7 to 9 sccm, implying that the etching of SiC is suppressed in the flow rate regime. The dramatic increase in the Si-O occupancy in this O_2_ flow rate regime played a major role in suppressing the SiC etch rate.

## 5. Conclusions

SiC etching in an HFE-347mmy/O_2_/Ar plasma was investigated at various flow rates of HFE-347mmy/O_2_. The etch rate of SiC increased, reached a maximum, and then decreased with an increase in the HFE-347mmy/O_2_ ratio. The etch rate behavior was elucidated in terms of factors either facilitating or suppressing the etching of SiC, i.e., the relative amounts of F and O radicals in favor of SiC etching and the thickness of the steady-state fluorocarbon film and Si-O occupancy against SiC etching.

When the flow rates of HFE-347mmy/O_2_ were varied from 3/7 to 9/1 sccm, the etch rate of SiC decreased because of a reduction in the amounts of F and O radicals, alongside an increase in the thickness of the steady-state fluorocarbon film. On the contrary, when the flow rates of HFE-347mmy/O_2_ were varied from 3/7 to 1/9 sccm, the etch rate of SiC decreased because the formation of SiO and SiO_2_ was dominant over SiC etching. Therefore, it is important to select appropriate flow rate conditions for effective SiC etching in the HFE-347mmy/O_2_/Ar plasma.

This study focused on the effect of varying discharge compositions on SiC etching in an HFE-347mmy/O_2_/Ar plasma. Future research may explore the systematic optimization of process parameters such as pressure, electrode temperature, and bias voltage to achieve higher etch rates and better surface quality.

## Figures and Tables

**Table 1:** Typical properties of HFE-347mmy.

Name	Heptafluoroisopropyl Methyl Ether (HFE-347mmy)
Molecular formula	C_4_H_3_F_7_O
Structural formula	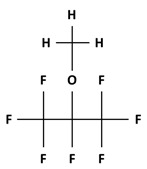
Boiling point	29 °C
Global warming potential	363

## Data Availability

Data are contained within the article.

## Nomenclature

g	gas phase
s	solid phase
